# Supplementary material for: Diet breadth and exploitation of exotic plants shift the core microbiome of Cephaloleia, a group of tropical herbivorous beetles
Source: PeerJ. 2018 May 17;6:e4793. doi: 10.7717/peerj.4793 (PMC5960584; doi:10.7717/peerj.4793)
Supplement: Supplemental Information 1 — All microbial relative abundance data was calculated from 16S rRNA barcode sequencing results. Heat scales are added for ease of visualization and are consistent among all columns. As is always the case in PCR-based analyses, relative abundance between taxa (i.e., comparing across a row) should be interpreted cautiously. A safer interpretation is the relative abundance of one taxon across multiple samples (i.e., comparing down a column). The full data set contains 274 OTUs, but these top 11 OTUs account for 48% of the total sequences recovered. [file peerj-06-4793-s001.pdf]

Table S1. Select key OTUs identified by SIMPER, representing notable taxonomic groups associated with six *Cephaloleia* species (n = 29 adult specimens and 5 eggs) - OTU#'s and colors match those on Figure 2.
All microbial relative abundance data was calculated from 16S rRNA barcode sequencing results. Heat scales are added for ease of visualization and are consistent among all columns.
As is always the case in PCR-based analyses, relative abundance between taxa (i.e., comparing across a row) should be interpreted cautiously. A safer interpretation is the relative abundance of one taxon across multiple samples (i.e., comparing down a column). The full data set contains 274 OTUs, but these top 11 OTUs account for 48% of the total sequences recovered.

				OTU 131476, 32% of all Pseudomonadales sequences	OTU 21817, 17% of all Pseudomonadales sequences	OTU 28305, 11% of all Pseudomonadales sequences	OTU 126400, 6% of all Pseudomonadales sequences	OTU 79811, 65% of all Enterobacteriales sequences	OTU 127346, 14% of all Enterobacteriales sequences	OTU 104860, 31% of all Burkholderiales sequences	OTU 7980, 63% of all Rickettsiales sequences	OTU 153335, 63% of all Rickettsiales sequences	OTU 57080, 100% of all Entomoplasmatales sequences	OTU 43304, 100% of all Legionellales sequences
			**Domain**	Bacteria	Bacteria	Bacteria	Bacteria	Bacteria	Bacteria	Bacteria	Bacteria	Bacteria	Bacteria	Bacteria
			**Phylum**	Proteobacteria	Proteobacteria	Proteobacteria	Proteobacteria	Proteobacteria	Proteobacteria	Proteobacteria	Proteobacteria	Proteobacteria	Tenericutes	Proteobacteria
			**Class**	Gammaproteobacteria	Gammaproteobacteria	Gammaproteobacteria	Gammaproteobacteria	Gammaproteobacteria	Gammaproteobacteria	Betaproteobacteria	Alphaproteobacteria	Alphaproteobacteria	Mollicutes	Gammaproteobacteria
			**Order**	Pseudomonadales	Pseudomonadales	Pseudomonadales	Pseudomonadales	Enterobacteriales	Enterobacteriales	Burkholderiales	Rickettsiales	Rickettsiales	Entomoplasmatales	Legionellales
			**Family**	Moraxellaceae	Moraxellaceae	Moraxellaceae	Pseudomonadaceae	Enterobacteriaceae	Enterobacteriaceae	Comamonadaceae	Rickettsiaceae	Rickettsiaceae	Spiroplasmataceae	Coxiellaceae
**Plant Type**	**Diet Breadth**	**Species**	**Genus**	*Acinetobacter*	*Acinetobacter*	*Acinetobacter*	*Pseudomonas*	undefined	undefined	undefined	*Rickettsia*	*Rickettsia*	*Spiroplasma*	*Rickettsiella*
**Native**	**Generalist**	**C. belti**	Cb1	1.0814	4.7728	0.1872	0.0208	2.2252	0.1976	0.5095	38.8999	0.0000	0.0000	0.0000
			Cb2	0.1005	0.0335	0.0112	0.0000	0.1787	0.0112	0.0000	35.4846	0.0000	0.0000	0.0000
			Cb3	0.2566	0.3894	0.0619	0.6991	0.4690	13.5487	0.0973	28.2566	0.0000	20.7788	0.0000
			Cb4	0.0317	0.0317	0.0317	0.0000	0.0952	0.0793	0.0000	40.6062	0.0000	28.5941	0.0000
			Cb5	0.2966	0.3838	0.4361	0.0000	0.3140	0.2094	0.0872	0.0000	0.0000	0.0000	0.0000
			Cb6	0.0254	0.0000	0.2715	0.0170	0.2036	0.0933	0.0000	2.1038	0.0000	8.4917	82.3549
			Cb7	4.0476	0.2086	3.2130	0.0313	1.6274	0.4486	0.0417	54.7152	0.0104	14.8237	0.4381
			Cb8	0.8086	1.0337	4.1931	1.5839	1.1337	0.0333	0.1417	0.0417	0.0000	0.9170	14.1047
		**C. reventazonica**	Cr1	4.0858	0.0311	0.0000	0.0000	3.8217	0.0000	0.0000	0.0466	27.6681	0.0000	0.0000
			Cr2	0.4431	0.0000	0.4009	0.0000	0.0211	0.0000	0.0000	0.0000	69.0863	0.0000	0.0000
			Cr3	1.0422	0.0183	0.0183	0.0000	0.4023	0.4937	0.0000	0.0183	14.2805	0.0000	0.0000
	**Speciialist**	**C. fenestrata**	Cf1	3.9437	0.9165	4.3881	0.1296	19.1261	1.8052	0.2222	0.0000	0.0000	0.0000	0.0000
			Cf2	3.5294	0.7843	2.5401	0.1783	7.6025	2.3529	0.1159	0.0000	0.0178	0.0000	0.0000
			Cf3	0.1083	0.0722	3.8503	0.4813	15.2930	21.3332	0.1203	0.0000	0.0000	0.0120	0.0000
			Cf4	0.0335	1.5757	30.2405	8.9682	5.5989	3.0676	0.4191	0.0000	0.0251	0.0000	0.0000
			Cf5	0.3041	5.8102	2.4652	1.9222	5.1260	1.3901	0.1738	0.0000	0.0000	0.0000	0.0000
			Cf6	0.9935	1.0266	17.7834	1.8876	1.1591	0.2870	0.4415	0.0000	4.5259	0.0000	0.0110
			Cf7	0.1254	1.5764	0.0717	0.0896	39.3820	0.0627	0.0179	0.0000	4.7559	0.6986	0.0358
		**C. dilaticollis**	Cdil1	18.8271	8.9269	1.0991	6.2254	39.6929	0.3188	1.7116	0.0084	0.0000	0.1594	0.0000
			Cdil2	27.5692	5.9286	1.1289	8.9556	27.9204	2.4417	2.0988	0.0000	0.0000	0.0836	0.0000
			Cdil3	9.5132	35.5800	3.4945	0.8586	7.3839	0.7470	1.8460	0.0172	0.0000	0.0601	0.0000
		**C. dorsalis**	Cdor1	0.0174	34.2078	1.1640	0.8947	4.8992	2.0413	2.5017	0.0521	0.0000	0.4083	0.0000
			Cdor2	0.0926	6.4019	2.1996	1.0882	5.4874	0.4052	1.7944	0.0000	0.0000	24.0912	0.0000
			Cdor3	0.4057	0.2525	0.3607	0.2615	21.1523	0.0451	2.4254	0.0000	0.0000	44.7119	0.0000
		**C. placida**	Cp1	37.5522	2.5052	2.4269	3.5491	7.3765	0.0435	6.3065	0.0000	0.0000	0.0087	0.0000
			Cp2	73.4161	0.2914	0.6580	1.6074	2.4629	2.1527	0.8460	0.0094	0.0000	0.0188	0.0000
			Cp3	3.5104	12.6863	1.8015	0.3620	0.4714	0.4209	0.2778	0.0000	0.0000	0.0000	0.0000
			Cp4	31.1617	0.1778	2.3198	0.8444	1.2799	0.5066	0.4000	0.0178	0.0000	0.0000	0.0000
			Cp5	24.8674	6.8931	1.1414	5.4642	11.6833	0.3056	15.0355	0.0090	0.0000	1.0785	0.0000
**Invasive**	**Generalist**	**C. belti**	Cb_inv1	0.2098	0.1049	0.1049	0.0000	0.2997	4.0012	0.0150	47.6098	0.0000	0.0300	0.0000
			Cb_inv2	0.0785	0.0000	0.0314	0.0000	0.0785	0.0157	0.0000	6.1244	0.0000	42.4152	0.0000
			Cb_inv3	0.3845	2.4665	0.6752	0.0188	1.9507	1.2754	0.0000	0.0000	0.0000	55.4534	0.0000
			Cb_inv4	0.0497	0.0000	0.3106	0.0124	3.4911	4.8081	0.0000	15.4056	0.0000	41.1852	0.0000
	**Speciialist**	**C. dilaticollis**	Cdil_inv2	0.0000	1.7655	4.6261	0.1322	0.9630	0.3965	0.0000	0.0000	0.0000	0.0094	0.0000
			Cdil_inv3	0.0000	16.7882	26.6857	0.9661	1.6759	0.2957	0.0000	0.0000	0.0000	0.0000	0.0000
			Cdil_inv1	0.0000	2.8591	4.3780	0.0199	1.5189	4.7255	0.0000	0.0000	0.0000	0.0099	0.0000
		**C. placida**	Cp_inv1	0.0262	16.4013	1.0409	0.5073	3.3852	0.0262	0.0000	0.0000	0.0000	0.0000	0.0000
			Cp_inv2	0.0378	21.2856	2.3976	0.6230	12.7808	0.0755	0.0094	0.0000	0.0189	0.0000	0.0566
**Lifestage**		**Species**												
**Eggs**	**Generalist**	**C. belti**	Cb_egg1	0.0227	24.8073	0.7029	1.9955	2.6304	0.7256	11.3379	0.0227	0.0000	0.0000	0.0000
			Cb_egg2	0.0000	9.1611	1.3690	0.8132	1.9763	0.2470	0.3911	0.0000	0.0200	0.0000	0.0000
	**Speciialist**	**C. dilaticollis**	Cdil_egg1	0.0000	12.0473	11.8886	3.6647	2.2940	0.3463	8.9742	0.0000	0.0000	0.0000	0.0000
			Cdil_egg2	4.4490	8.5421	2.4641	0.8761	7.0910	1.0130	0.3285	0.0000	0.0300	0.0000	0.0000
		**C. dorsalis**	Cdor_egg	0.0894	13.9013	3.6742	9.8248	4.9884	23.9764	1.7343	0.0000	0.0000	0.0179	0.0000
